# lncRNA-CASC15 promotes osteosarcoma proliferation and metastasis by regulating epithelial-mesenchymal transition via the Wnt/β-catenin signaling pathway

**DOI:** 10.3892/or.2022.8278

**Published:** 2022-02-01

**Authors:** Hongqi Wang, Peng Zhang

Oncol Rep 45: Article no. 76, 2021; DOI: 10.3892/or.2021.8027

Following the publication of this article, the authors have re-examined their raw data and realized that the data of each group in Fig. 5B were inadvertently mixed up when the statistical analysis was performed, resulting in inconsistencies comparing between the presented results and the corrresponding results in Fig. 5A. Furthermore, the authors also realized that the expression levels of some of the genes had not been standardized.

A corrected version of Fig. 5, showing more representative data for the vimentin and cyclin D blots in Fig. 5A and the corrected statistical analysis for Fig. 5B, is shown below. The authors sincerely apologize for the errors that went unnoticed before their paper was published, and thank the Editor for allowing them the opportunity to publish a Corrigendum. They also regret any inconvenience that these mistakes may have caused.

## Figures and Tables

**Figure 5:**
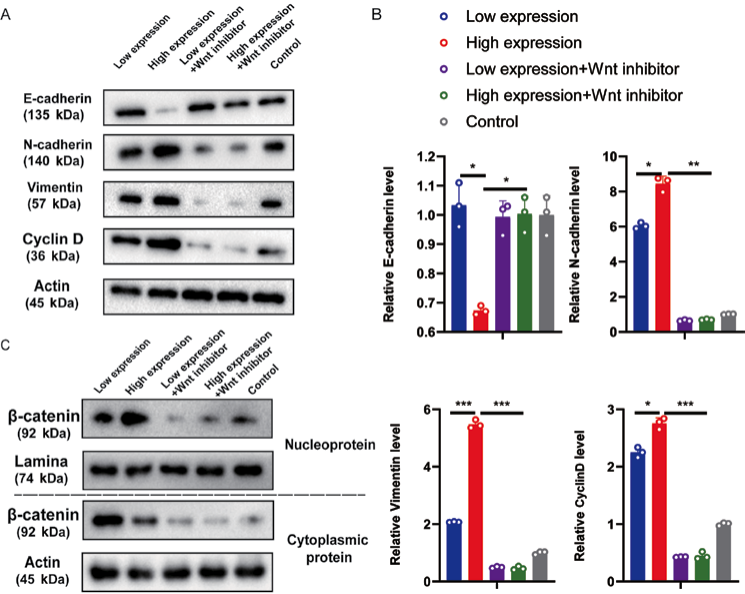
CASC15 promotes the epithelial-mesenchymal transition of osteosarcoma cells by Wnt/β-catenin pathway. U-2OS cells were transfected with 1 ng/well of oe-CASC15 as the high expression dose, and 0.6 ng/well as the low expression dose. The transfection groups were as follows: high expression, low expression, high expression + Wnt inhibitor, and low expression + Wnt inhibitor. (A and B) Reverse transcription-quantitative polymerase chain reaction and western blotting were used to detect the expression of E-cadherin, N-cadherin, vimentin, and cyclin D, respectively. (C) After nuclear and cytoplasmic separation, western blotting was used to detect the expression changes of β-catenin among the aforementioned groups. ASC15, cancer susceptibility 15.

